# Supplementary material for: Investigating the Modes of Action of the Antimicrobial Chalcones BC1 and T9A
Source: Molecules. 2020 Oct 9;25(20):4596. doi: 10.3390/molecules25204596 (PMC7587203; doi:10.3390/molecules25204596)
Supplement: Supplementary file 1 [file molecules-25-04596-s001.pdf]

Supplementary Material

Investigating the Modes of Action of the Antimicrobial Chalcones BC1 and T9A

Luana G. Morão [1,†], André S. G. Lorenzoni [2,†], Parichita Chakraborty [2], Gabriela M. Ayusso [3], Lucia B. Cavalca [2], Mariana B. Santos [3], Beatriz C. Marques [3], Guilherme Dilarri [1], Caio Zamuner [1], Luis O. Regasini [3], Henrique Ferreira [1,*] and Dirk-Jan Scheffers [2,*]

[1] Departamento de Bioquímica e Microbiologia, Instituto de Biociências, Universidade Estadual Paulista, 130506-900 SP, Rio Claro, Brazil; luanagm_bio@yahoo.com.br (L.G.M.); gui_dila@hotmail.com (G.D.); caiozamuner@gmail.com (C.Z.)

[2] Department of Molecular Microbiology, Groningen Biomolecular Sciences and Biotechnology Institute, University of Groningen, 9747 AG Groningen, The Netherlands; andreglock@gmail.com (A.S.G.L.); p.chakraborty@rug.nl (P.C.); l.bonci.cavalca@rug.nl (L.B.C.)

[3] Departamento de Química e Ciências Ambientais, Instituto de Biociências, Letras e Ciências Exatas, Universidade Estadual Paulista, 15054-000 SP, São José do Rio Preto, Brazil; gabimayusso@gmail.com (G.M.A.); mariana19bsantos@gmail.com (M.B.S.); bbiacarvalho@outlook.com (B.C.M.); luis.regasini@unesp.br (L.O.R.)

* Correspondence: henrique.ferreira@unesp.br (H.F.); d.j.scheffers@rug.nl (D.-J.S.); Tel.: +31-50-3632319 (D.-J.S.)

† These authors contributed equally to this work.

Table 1. Structures and minimum inhibitory concentrations of hydroxychalcones synthesized and tested by Ayusso and Regasini (unpublished results). MIC_{50} is the concentration necessary to cause 50 % decrease in resazurin reduction by *S. aureus* (Sa) or *P. aeruginosa* (Pa) cells after 24 hours in the presence of the compound, values displayed are in μg/mL.

Compound	MIC_{50} Sa	MIC_{50} Pa	R	R′
**3**	12.5	50.0	3′-hydroxyphenyl	phenyl
**4**	50.0	100	4′-hydroxyphenyl	phenyl
**5 (T9A)**	6.2	100	phenyl	2-hydroxyphenyl
**6**	25.0	50.0	phenyl	3-hydroxyphenyl
**vancomycin**	2.0	-	-	-

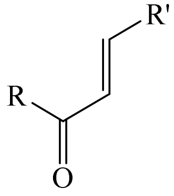

**gentamicin**	-	4.0	-	-

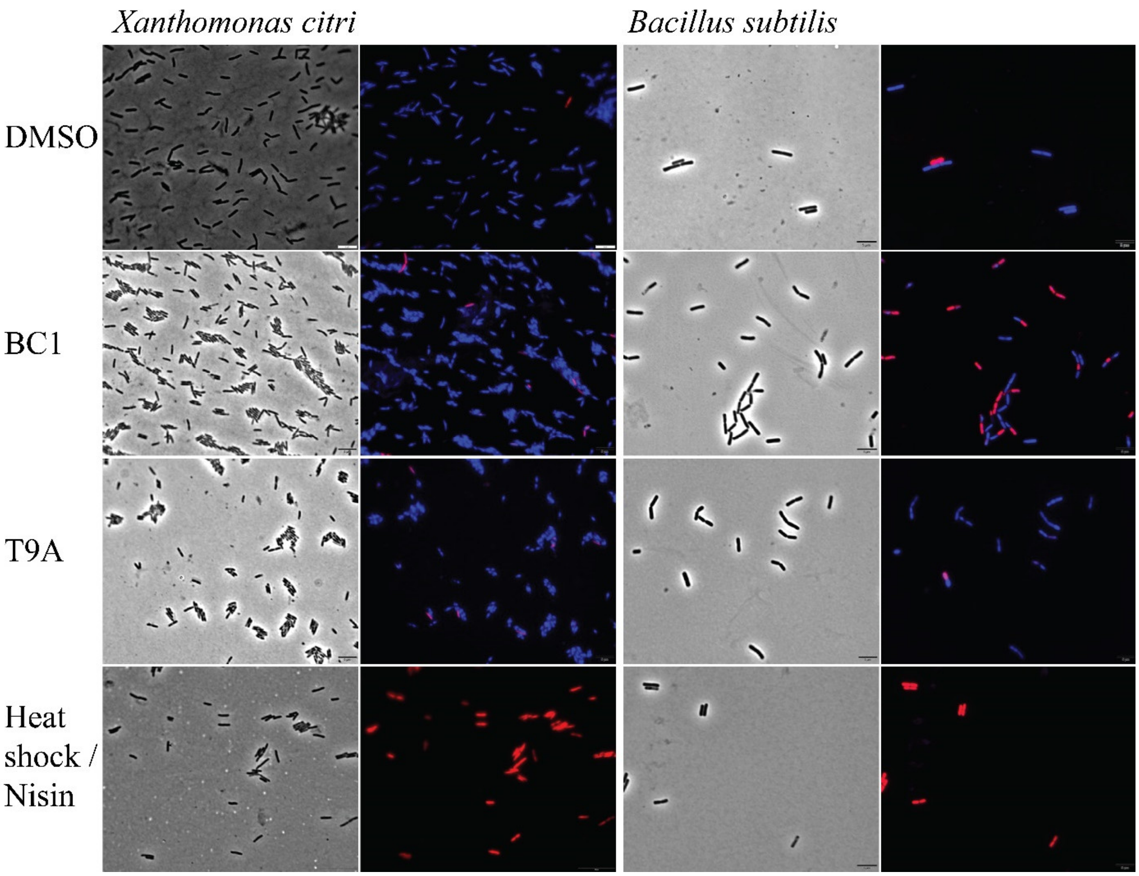

Figure S1. Membrane permeability assay. *X. citri* and *B. subtilis* cells were exposed to the compounds as indicated on the left-hand side of the panels for 15 min, and after stained with Propidium iodide (PI) and SYTO 9. Phase contrast (left) and overlay of Texas Red and DAPI (right). Magnification 100X; scale bars correspond to 5 µm.

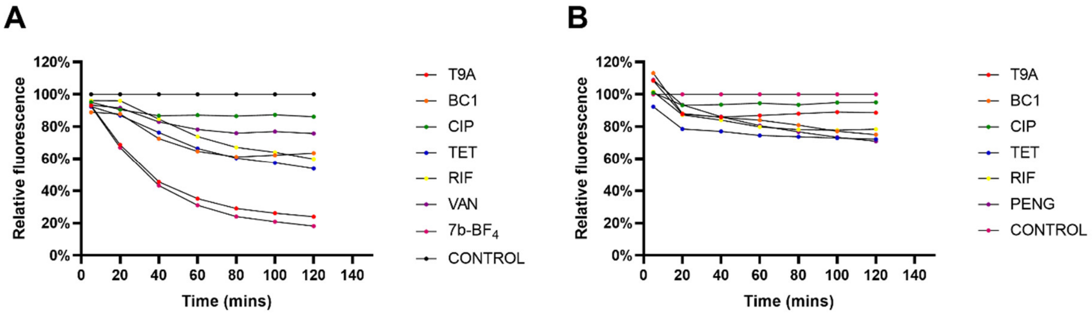

Figure S2. Relative inhibition by compounds in the REMA assay. Data from Fig 3 are plotted as relative to control with the fluorescence of the control sample at each time point set to 100% and the relative fluorescence calculated for each treatment at each individual timepoint. Panel A: *B. subtilis* cells; panel B: *X.citri* cells.
